# Anti-inflammatory effect of rebamipide on the ocular surface

**DOI:** 10.1186/2045-7022-3-S2-P21

**Published:** 2013-07-16

**Authors:** Mayumi Ueta, Chie Sotosono, Norihiko Yokoi, Shigeru Kinoshita

**Affiliations:** 1 Doshisha University, Kyoto Prefectural University of Medicine, Uji, Japan; 2 Kyoto Prefectural University of Medicine, Ophthalmology, Kyoto, Japan

## Purpose

The eyedrop form of rebamipide was approved in Japan for use in the treatment of dry eye diseases, because it up-regulates mucin secretion and production. Others reported that rebamipide, a gastroprotective drug, could not only increase gastric mucus production but also suppressed gastric mucosal inflammation and that it was dominantly distributed in mucosal tissues. In this study we investigated whether rebamipide has anti- inflammatory effects in the ocular surface.

## Methods

We used ELISA and quantitative RT-PCR assay to examine the effects of rebamipide on polyI:C-induced cytokine expression by primary human conjunctival epithelial cells. We studied the effects of rebamipide on ocular surface inflammation in our murine experimental allergic conjunctivitis (EAC) model. Moreover, we also observed their condition of allergic conjunctivitis when we treated the dry eye patients accompanied with allergic conjunctivitis using rebamipide.

## Results

Rebamipide could suppress polyI:C-induced cytokine mRNA expression and the production of CXCL10, CXCL11, RANTES, MCP-1, and IL-6 in human conjunctival epithelial cells. The topical administration of rebamipide suppressed conjunctival allergic eosinophil infiltration in our EAC model. Furthermore, the allergic conjunctivitis which accompanied by dry eye patients treated with rebamipide tended to be better.

## Conclusions

The topical application of rebamipide on the ocular surface might suppress ocular surface inflammation by suppressing the production of cytokines by ocular surface epithelial cells.

